# The first wave of the COVID-19 pandemic in Spain: characterisation of cases and risk factors for severe outcomes, as at 27 April 2020

**DOI:** 10.2807/1560-7917.ES.2020.25.50.2001431

**Published:** 2020-12-17

**Authors:** Working group for the surveillance and control of COVID-19 in Spain, Members of the Working group for the surveillance and control of COVID-19 in Spain, Members of the Working group for the surveillance and control of COVID-19 in Spain, Lidia Redondo-Bravo, María José Sierra Moros, Elena Vanessa Martínez Sánchez, Nicola Lorusso, Alberto Carmona Ubago, Virtudes Gallardo García, Pilar Sánchez Villanueva, Adela Puy Azón, Joaquín Guimbao Bescós, An LD Boone, Ana Fernández Ibáñez, Blanca Álvarez Fernández, Antonio Nicolau Riutort, Magdalena Salom Castell, Jaume Giménez Duran, Domingo Núñez Gallo, Magdalena Lucia Rojo Moreno, Aniceto Blasco de la Fuente, Luis Javier Viloria Raymundo, Marcos Hernández Pereña, Matilde Chico Mena, Sonia Humanes Aparicio, Maria Soledad Illescas Fernández, Socorro Fernández Arribas, Mª del Henar Marcos Rodríguez, Isabel Martínez-Pino, Mireia Jané, Ana Martínez, Pilar Ciruela, Katja Villatoro Bongiorno, Aina March Yagüe, Jordi Pérez Panadés, María del Mar López-Tercero Torvisco, Cecilia Gordillo Romero, Beatriz Caleya Olivas, Sara De Miguel García, Esther Córdoba Deorador, Elisa Gil Montalbán, Manuel del Valle Arrojo, Luisa Abraira García, Antonio Boullosa Cortés, Ana García-Fulgueiras, Mª Dolores Chirlaque, Mª Isabel Barranco, Itziar Casado, Jesús Castilla, Manuel García-Cenoz, Fernando Gonzalez Carril, Amaia Soraluce Olañeta, Mª Jesús Lázaro-Carrasco de la Fuente, Eva Martínez Ochoa, Ana Carmen Ibáñez Pérez, Ángela Blanco Martínez, Ana Isabel Rivas Pérez, Violeta Ramos Marín, Margarita Medina Vinuesa, Daniel Castrillejo Pérez, Atanasio Alfonso Gómez Anés, Francisco Pozo, Inmaculada Casas, Belén Peñalver-Argüeso, Despina Pampaka, Jesús A. Oliva Domínguez, María Sastre García, Amparo Larrauri

**Affiliations:** 1 The members of the Working group are listed at the end of the article

**Keywords:** Spain, COVID-19, SARS-CoV-2, surveillance, pandemic, risk factors

## Abstract

**Background:**

The first wave of the coronavirus disease (COVID-19) pandemic spread rapidly in Spain, one of Europe’s most affected countries. A national lockdown was implemented on 15 March 2020.

**Aim:**

To describe reported cases and the impact of national lockdown, and to identify disease severity risk factors.

**Methods:**

National surveillance data were used to describe PCR-confirmed cases as at 27 April 2020. We compared case characteristics by severity categories (hospitalisation, admission to intensive care unit (ICU), death) and identified severity risk factors using multivariable regression.

**Results:**

The epidemic peaked on 20 March. Of 218,652 COVID-19 cases, 45.4% were hospitalised, 4.6% were admitted to ICU and 11.9% died. Among those who died, 94.8% had at least one underlying disease. Healthcare workers (HCWs) represented 22.9% of cases. Males were more likely to have severe outcomes than females. Cardiovascular disease was a consistent risk factor. Patients with pneumonia had higher odds of hospitalisation (odds ratio (OR): 26.63; 95% confidence interval (CI): 25.03–28.33). The strongest predictor of death was age ≥ 80 years (OR: 28.4; 95% CI: 19.85–40.78). Among underlying diseases, chronic renal disease had highest odds of death (OR: 1.47; 95% CI: 1.29–1.68).

**Conclusions:**

COVID-19 case numbers began declining 6 days after the national lockdown. The first wave of the COVID-19 pandemic in Spain had a severe impact on elderly people. Patients with cardiovascular or renal conditions were at higher risk for severe outcomes. A high proportion of cases were HCWs. Enhanced surveillance and control measures in these subgroups are crucial during future COVID-19 waves.

## Introduction

As at 27 April 2020, 2,878,196 confirmed coronavirus disease (COVID-19) cases were notified globally, including 198,668 deaths, of which 1,359,380 cases and 124,525 deaths occurred in Europe [1]. Spain and Italy were, at that time, the epicentres of the COVID-19 pandemic in Europe. Spain experienced the highest number of cases, while Italy observed the highest number of deaths [2].

The initial escalation of the situation in China and the confirmation of cases outside of China prompted countries to set up surveillance systems for early detection of SARS-CoV-2 infections, as part of preparedness efforts. A national protocol for the management of COVID-19 cases that included surveillance guidelines was developed by Spain’s Ministry of Health and the Autonomous Regions (ARs) a few days after the World Health Organization (WHO) mission in China confirmed human-to-human transmission of COVID-19 on 22 January 2020 [3,4]. National universal surveillance of confirmed COVID-19 cases of any severity was implemented. Following the protocol, the first imported case in Spain was detected in La Gomera, in the Canary Islands, on 31 January, while the first case of locally acquired COVID-19 was confirmed on 26 February [5,6]. COVID-19 cases with symptom onset before the first local transmission case on 26 February were retrospectively identified in samples swabbed for other diagnostic purposes or in hospitalised cases with pneumonia who were posteriorly screened for COVID-19 after a change in testing policy (unpublished data).

The protocol was dynamic and subject to changes based on the evolution of the pandemic, as well as the priorities and response capacities at any particular time. Priorities included measures aimed at early detection of cases, their immediate diagnosis and isolation, as well as identification and monitoring of contacts with syndromic and virological surveillance. Almost 220,000 confirmed COVID-19 cases were notified to the National Epidemiological Surveillance Network (RENAVE in Spanish) as at 27 April.

The aim of this work was to describe the main epidemiological and clinical characteristics of COVID-19 cases and the impact of the national lockdown on the first wave of the pandemic, with a view of gaining further understanding of the epidemiological situation in Spain.

## Methods

COVID-19 surveillance in Spain was supported by the RENAVE’s activities. This network is in charge of national surveillance of communicable infectious disease and outbreaks. The RENAVE is managed by the National Centre of Epidemiology (CNE in Spanish), while all public health responses and actions derived from the surveillance information are coordinated by the Centre of Health Alerts and Emergencies (CCAES in Spanish) at the Ministry of Health. The CNE runs the Spanish Surveillance System electronic platform (SiViES in Spanish) that facilitates electronic reporting, validation and data management of cases reported by the Regions (17 ARs and two Autonomous Cities).

### COVID-19 case definition

According to the first protocol, a case under investigation was a person meeting both epidemiological and clinical criteria. The epidemiological criteria included a history of travel to COVID-19–affected areas or epidemiological links with COVID-19 laboratory-confirmed cases. The clinical criteria included symptoms compatible with a severe acute respiratory infection (SARI) and evidence of pneumonia (clinical or radiological), or fever and symptoms of an acute respiratory infection (ARI) such as dyspnoea, cough or sore throat.

A confirmed case was defined as any person with laboratory confirmation of SARS-CoV-2 infection by RT-PCR test. A probable case was defined as a suspected case for whom testing for SARS-CoV-2 was performed, but the results reported by the laboratory were inconclusive.

### Testing algorithms

Since the beginning of the epidemic in Spain, universal notification of COVID-19 cases tested by reverse-transcription PCR (RT-PCR) was mandatory and urgent for hospitalised and non-hospitalised cases. The case definition and the testing strategy were dynamic throughout the first wave. Testing was initially prioritised for patients with ARI symptoms and epidemiological links or severe clinical presentation, but during the epidemic’s peak diagnostic prioritisation changed to severe cases and essential services workers. Rapid antibody and ELISA-based serological tests were introduced in April to test and screen for COVID-19 in non-hospital settings.

### Data collection

Samples, as well as clinical and epidemiological information, were collected at a regional level and data were entered electronically in SiViES. Only confirmed and probable cases were reported to the RENAVE.

A standardised clinical form was used to collect data, including information on: demographics (sex, age), clinical presentation (fever, cough, sore throat, shortness of breath, myalgia, vomiting, diarrhoea), epidemiological risk factors (contact with a COVID-19 case, person with ARI, healthcare worker (HCW)), clinical information (hospitalisation, pneumonia, acute respiratory distress syndrome (ARDS), acute renal failure, intensive care unit (ICU) admission, mechanical ventilation), comorbidities (cardiovascular and respiratory diseases, diabetes, arterial hypertension, chronic kidney disease, other chronic diseases), date of symptom onset, date of diagnosis, date of notification to the AR, outcome, region of diagnosis and province of residence.

### Definition of healthcare workers and clinical severity

The cases were categorised as HCW if they had active employment in the healthcare sector, regardless of their role.

Clinical severity of COVID-19 cases was classified in the following categories: (i) no hospitalisation, (ii) hospitalisation, (iii) ICU admission and (iv) death (any case who died and was infected with SARS-CoV-2). These categories are not mutually exclusive and the total can add up more than 100%, as cases admitted to an ICU were also included in ‘hospitalisation’ and deaths were included in ‘no hospitalisation’ or ‘hospitalisation’.

### Data analysis

The analysis was restricted to COVID-19 cases confirmed by PCR, with onset of symptoms from 31 January to 27 April, when lockdown restrictions were eased.

When the symptom onset date was missing, it was estimated using the date of diagnosis minus 6 days (median of days between symptom onset and date of diagnostic in this sample) (Table 1). The cases confirmed only by serological tests were excluded from the analysis. Missing diagnostic tests were assumed to be PCR if the date of diagnosis was before 8 April, when the protocol on the use of rapid antibody and ELISA-based serological tests was published. All percentages were calculated using the number of patients with available data for a specific variable as the denominator. Crude case fatality rates (CFR) were calculated as the total number of COVID-19 deaths divided by the total number of cases.

**Table 1:** Demographic, clinical and epidemiological characteristics of COVID-19 cases, RENAVE, Spain, January–April 2020 (n = 218,652)

Characteristics	Total		Females		Males		p value^a^
	n	%	n	%	n	%	
Sex	NA	NA	122,870	56.2	95,769	43.8	NA
Age, median (IQR)	61	46–78	59	44–80	62	48–77	< 0.001
**Age group (years)**							
< 2	291	0.1	119	0.1	172	0.2	NA
2–4	133	0.1	63	0.1	70	0.1	NA
5–14	614	0.3	293	0.2	321	0.3	NA
15–29	13,119	6.0	8,701	7.1	4,418	4.6	NA
30–39	20,438	9.4	12,813	10.4	7,625	8.0	NA
40–49	31,806	14.6	18,607	15.1	13,199	13.8	NA
50–59	38,645	17.7	21,787	17.7	16,858	17.6	NA
60–69	31,679	14.5	15,026	12.2	16,652	17.4	NA
70–79	30,704	14.0	13,645	11.1	17,058	17.8	NA
≥ 80	51,144	23.4	31,792	25.9	19,352	20.2	< 0.001
**Clinical presentation^b^**							
Fever	72,006	74.4	35,935	69.4	36,069	80.1	< 0.001
Cough	63,367	69.6	33,504	68.3	29,861	71.3	< 0.001
Sore throat	7,685	21.0	4,942	23.8	2,742	17.3	< 0.001
Shortness of breath	41,050	48.4	20,165	44.4	20,883	53.0	< 0.001
Shivering	8,064	22.1	4,419	21.4	3,644	23.0	< 0.001
Vomiting	3,040	8.4	1,985	9.8	1,055	6.7	< 0.001
Diarrhoea	9,893	26.1	5,816	27.2	4,077	24.8	< 0.001
Pneumonia	66,540	56.3	28,974	46.9	37,565	66.7	< 0.001
Acute respiratory distress syndrome	6,055	7.4	2,288	5.2	3,766	10.1	< 0.001
Other respiratory symptoms	6,701	9.8	2,819	7.6	3,882	12.4	< 0.001
Acute renal failure	4,635	5.7	1,748	4.0	2,887	7.9	< 0.001
**Comorbidities**							
One or more	92,717	65.6	47,763	62.0	44,954	69.8	< 0.001
Cardiovascular disease	40,625	30.6	18,832	26.1	21,793	35.9	< 0.001
Chronic lung disease	15,354	11.6	6,504	9.0	8,850	14.6	< 0.001
Chronic renal disease	3,335	2.5	1,681	2.3	1,654	2.7	< 0.001
Diabetes	22,214	16.7	10,027	13.9	12,187	20.1	< 0.001
Hypertension^c^	25,529	19.2	13,517	18.7	12,012	19.8	< 0.001
**Severity**							
Hospital admission	89,631	45.4	39,827	36.1	49,803	57.3	< 0.001
Mechanical ventilation	5,297	7.1	1,626	4.3	3,671	10.2	< 0.001
ICU admission	8,289	4.6	2,542	2.6	5,747	7.2	< 0.001
Death	26,121	11.9	11,441	9.3	14,680	15.3	< 0.001
**Risk exposure**							
Close contact with COVID-19 case	8,359	54.6	5,301	60.5	3,058	46.6	< 0.001
Contact with person with ARI	6,725	51.5	4,197	57.2	2,528	44.2	< 0.001
Healthcare worker	36,153	22.9	27,682	31.2	8,471	12.3	< 0.001
**Relevant time lags (days), median (IQR)**							
Symptom onset to diagnosis	6	3–10	6	3–11	7	3–10	< 0.001
Symptom onset to hospital admission	7	3–10	7	3–10	7	3–10	< 0.001
Symptom onset to ICU admission	9	6–12	9	6–12	9	6–12	0.169
Symptom onset to death	15	9–25	15	9–25	15	9–25	< 0.001

ARI: acute respiratory infection; COVID-19: coronavirus disease; ICU: intensive care unit; IQR: interquartile range; NA: not applicable; RENAVE: National Epidemiological Surveillance Network.

^a^ p value for the comparison of females and males.

^b^ Percentages are calculated on COVID-19 cases with available information on that variable.

^c^ Data on hypertension were collected as of 18 March.

An epidemic curve was plotted with the number of confirmed cases by date of symptom onset. Cumulative incidence rates by AR and province were calculated using the 2019 population estimates available from the Spanish National Institute of Statistics [7] and were adjusted for age.

Multivariable logistic regression models were applied to identify possible independent risk factors for severe outcomes (hospitalisation, ICU admission and death), restricted to hospitalised cases. The factors examined included sex; age group; presence of pneumonia, ARDS, acute renal failure or other underlying diseases (cardiovascular disease, chronic lung disease, diabetes, hypertension or chronic kidney disease); time in days from onset of symptoms to diagnosis; time in days from onset of symptoms to death and whether or not they were a HCW. Apart from demographics, these variables were selected based on a literature review, factors that had a biological link with the outcome and variables that were not incorporated or reflected in other factors. The model was also adjusted by AR, date of diagnosis and date of notification to the AR. Adjusted odds ratios (ORs) and 95% confidence intervals (CIs) were estimated. In order to study any differences by sex, we ran the model separately for each severe outcome (hospitalisation, ICU admission and death). The analyses were performed using Stata 15.1 (Stata Corp, College Station, United States (US)).

### Ethical statement

The surveillance protocol was approved by the Spanish Inter-territorial Council of the National Health System. This is a rector group that ensures that public health measures are carried out in all the ARs with a vision of equity.

Our study was part of the public health response to the outbreak, and therefore no explicit ethical evaluation was necessary. Although individual informed consent was not required, all data were pseudonymised to protect patient privacy and confidentiality.

## Results

**Figure 1:**
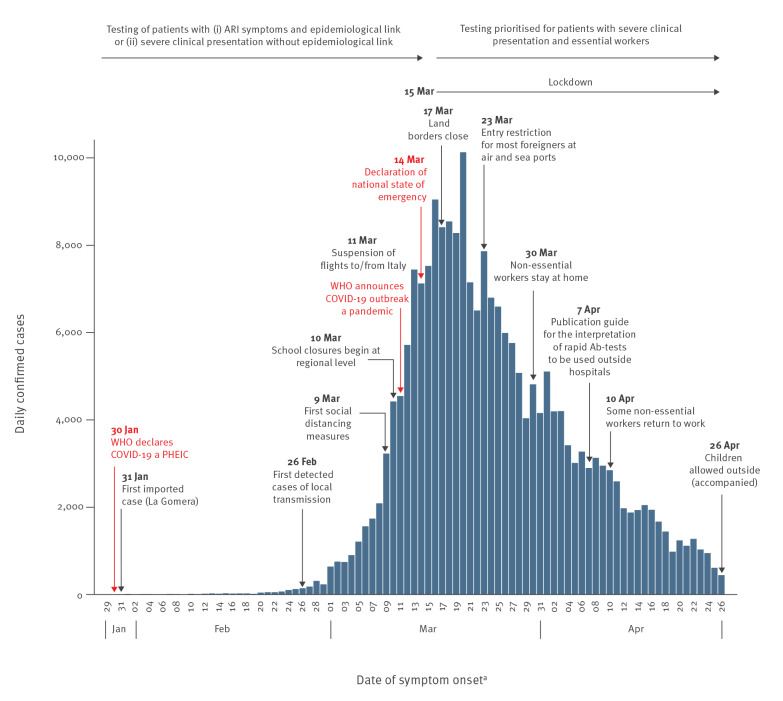
Number of confirmed COVID-19 cases and relevant national and WHO announcements, RENAVE, Spain, January–April 2020 (n = 218,652)

ARI: acute respiratory infection; Ab-test: diagnostic test based on antibody detection; COVID-19: coronavirus disease; PHEIC: Public Health Emergency of International Concern; RENAVE: National Epidemiological Surveillance Network; WHO: World Health Organization.

^a^ For cases missing a date of symptom onset, date of diagnosis minus 6 days was used.

The evolution of the first wave of the COVID-19 pandemic in Spain, as well as the dates of national and international events and public health measures, are shown in Figure 1. Daily number of COVID-19 cases by onset of symptoms peaked on 20 March and steadily decreased after this date, 6 days after the national lockdown was implemented. The distribution of COVID-19 cases by severity over time evidenced that the highest number of daily admissions to hospital or ICU was observed around 24 March, while deaths presented a plateau for a longer time (Supplementary Figure S1).

Red text indicates the most relevant institutional announcements.

### Geographical distribution

**Figure 2:**
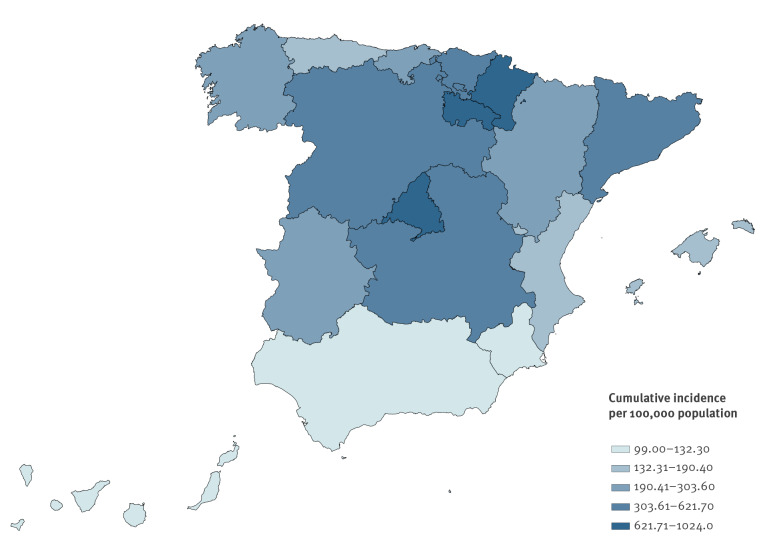
Age-adjusted cumulative incidence of COVID-19 cases by Autonomous Region, RENAVE, Spain, January–April 2020

COVID-19: coronavirus disease; RENAVE: National Epidemiological Surveillance Network.

Adjusted cumulative incidence ranged from 99 cases per 100,000 population in the AR of Murcia to 1,024 in the AR of La Rioja (Figure 2). The highest adjusted cumulative incidence rates were recorded in Navarre, Madrid and La Rioja (632.6–1,024/100,000 population). Differences in the adjusted cumulative incidence of provinces within the same AR are shown in Supplementary Figure S2. Maximum cumulative incidences were reached in provinces belonging to the most affected AR.

### Demographic and clinical characteristics and underlying diseases

From 31 January to 27 April, a total of 218,652 COVID-19 cases were notified to the RENAVE. Of these, 45.4% (n = 89,631) were hospitalised, 4.6% (n = 8,289) were admitted to ICU and 11.9% (n = 26,121) died.

The median age of COVID-19 cases was 61 years old (interquartile range (IQR): 46–78), higher for males than for females (62 vs 59), and 84.2% of cases were ≥ 40 years old. Females were 56.2% of the total number of cases. The overrepresentation of women was observed in the 15–49 years and ≥ 80 years age groups (Table 1).

Fever (74.4%), cough (69.6%) and shortness of breath (48.4%) were the most prevalent symptoms in both women and men (Table 1). Sore throat and gastrointestinal symptoms were more frequent in females than in males. Clinical complications such as pneumonia, ARDS and acute renal failure were significantly more common among men.

Two thirds of cases reported at least one chronic condition, the most frequent being cardiovascular disease (30.6%), followed by hypertension (19.2%). Underlying diseases considered in the study were more prevalent among males than females (Table 1).

**Figure 3:**
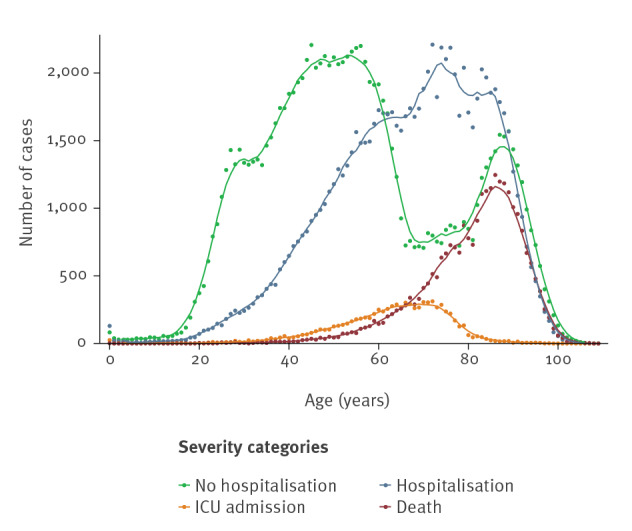
Distribution of COVID-19 cases by age and severity categories, RENAVE, Spain, January–April 2020

COVID-19: coronavirus disease; ICU: intensive care unit; RENAVE: National Epidemiological Surveillance Network.

Almost half of the hospitalised cases (49.1%) were ≥ 70 years old (Table 2). ICU admission was highest in 60–79 year olds (61.0%) and decreased to 4.7% in patients ≥ 80 years old. On the contrary, 62.0% of deaths were reported among patients aged ≥ 80 years and only 5.1% of deaths occurred in patients < 60 years old. The distribution of cases by age and severity categories is further explored in Figure 3, which shows that non-hospitalised cases had a bimodal distribution, with the first peak observed between 50–60 years old and the second around 90 years old.

**Table 2:** Demographic and clinical characteristics of confirmed COVID-19 cases by severity categories, RENAVE, Spain, January–April 2020

Characteristics	No hospitalisation		Hospitalisation		ICU admission		Death	
	n	%	n	%	n	%	n	%
Total	107,713	54.6	89,631	45.4	8,289	4.6	26,121	11.9
**Sex**								
Females	70,579	65.5	39,827	44.4	2,542	30.7	11,441	43.8
Males	37,133	34.5	49,803	55.6	5,747	69.3	14,680	56.2
**Age, median (IQR)**	53 (55–80)	NA	69 (40–72)	NA	6 4 (40–72)	NA	83 (75–89)	NA
**Age group (years)**								
< 2	121	0.1	150	0.2	26	0.3	2	0.0
2–4	83	0.1	39	0.0	4	0.0	0	0.0
5–14	417	0.4	130	0.1	22	0.3	3	0.0
15–29	10,402	9.7	1,694	1.9	100	1.2	46	0.2
30–39	14,777	13.7	3,925	4.4	279	3.4	82	0.3
40–49	20,190	18.7	8,747	9.8	746	9.0	285	1.1
50–59	20,854	19.4	14,085	15.7	1,668	20.1	919	3.5
60–69	11,925	11.1	16,848	18.8	2,695	32.5	2,457	9.4
70–79	8,008	7.4	20,117	22.4	2,363	28.5	6,141	23.5
≥ 80	20,915	19.4	23,889	26.7	386	4.7	16,185	62.0
**Chronic diseases**								
One or more	29,606	49.4	54,305	79.1	5,470	81.2	19,085	94.8
Cardiovascular disease	10,064	17.7	27,659	42.2	2,676	43.3	11,444	59.9
Chronic lung disease	4,063	7.1	9,961	15.2	961	15.5	3,960	20.7
Diabetes	5,050	8.9	15,096	23.1	1,587	25.7	6,207	32.5
Hypertension	6,432	11.3	15,303	23.4	1,065	17.2	6,514	34.1
Chronic renal disease	1,056	1.9	2,253	3.4	166	2.7	1,166	6.1
Pneumonia	5,988	13.8	56,432	83.7	6,123	91.0	14,305	81.2
**Other**								
Mechanical ventilation	56	0.2	4,927	10.9	4,085	77.9	2,060	16.8
Healthcare worker	29,217	38.4	3,981	5.8	310	4.9	109	0.6

COVID-19: coronavirus disease; ICU: intensive care unit; IQR: interquartile range; NA: not applicable; RENAVE: National Epidemiological Surveillance Network.

Percentages were calculated excluding cases with missing values. Severity categories are not exclusive; cases admitted to ICU are also included in ‘hospitalisation’ and deaths are also included in ‘no hospitalisation’ or ‘hospitalisation’.

Age distribution was similar among men and women, with the majority of HCW cases in the age group 40–59 years (54%).

The majority of non-hospitalised cases were females (65.5%), whereas most of the patients with a severe outcome (hospitalisation, admission to ICU, death) were males (55.6%, 69.3% and 56.2%, respectively). Among patients who died, 94.8% had at least one underlying disease (Table 2). Cardiovascular disease was the most frequent comorbidity for all the severity categories, especially among the deceased (59.9%). Pneumonia was also more frequent among the patients included in the severe outcome categories. Nine in 10 patients (91.0%) admitted to an ICU presented with pneumonia and 77.9% required mechanical ventilation (Table 2).

The median time interval between onset of symptoms and diagnosis was 6 days (IQR: 3–10) and from onset of symptoms to death was 15 days (IQR: 9–25) (Table 1).

### Exposure variables

Of those cases that had information about potential exposures to SARS-CoV-2, 54.6% reported close contact with a COVID-19 case and 51.5% had contact with a person with ARI (Table 1). One in five cases (22.9%) was a HCW and, among them, 77% were females (Table 1).

### Risk factors for hospitalisation, intensive care unit admission and death

The results of the multivariable logistic regression models are shown in Table 3. All the factors studied (sex, age, pneumonia, ARDS, chronic conditions, time from symptom onset to diagnosis and HCW) were associated with hospitalisation and death.

**Table 3:** Demographic and clinical factors associated with clinical severity of COVID-19, RENAVE, Spain, January–April 2020

Characteristics	Hospitalisation				ICU admission^a^				Death^a^			
	Hosp	Non-hosp	OR (95% CI)	p value	ICU	No ICU	OR (95% CI)	p value	Died	Recovered	OR (95% CI)	p value
**Sex**												
Females	39,827	70,579	1.00	< 0.001	2,367	34,585	1.00	< 0.001	7,666	32,161	1.00	< 0.001
Males	49,803	37,133	1.42 (1.34–1.50)		5,338	41,327	1.36 (1.24–1.51)		11,528	38,275	1.33 (1.24–1.42)	
**Age group (years)**												
< 40	5,938	25,800	1.00	< 0.001	391	5,184	1.00	< 0.001	104	5,834	1.00	< 0.001
40–59	22,832	41,044	1.46 (1.33–1.59)		2,258	19,048	1.31 (1.06–1.63)		1,021	21,811	1.91 (1.32–2.77)	
60–69	16,848	11,925	2.78 (2.50–3.08)		2,496	13,234	1.79 (1.44–2.24)		2,102	14,746	4.84 (3.37–6.96)	
70–79	20,117	8,008	4.56 (4.06–5.12)		2,197	16,715	1.24 (0.99–1.56)		5,126	14,991	10.01 (6.99–14.35)	
≥ 80	23,889	20,915	3.48 (3.11–3.90)		363	21,729	0.12 (0.09–0.16)		10,841	13,048	28.45 (19.85–40.78)	
**Pneumonia**												
No	11,012	37,559	1.00	< 0.001	555	9,469	1.00	< 0.001	2,028	8,984	1.00	< 0.001
Yes	56,432	5,988	26.63 (25.03–28.33)		5,760	47,140	1.50 (1.30–1.73)		12,814	43,618	1.24 (1.13–1.35)	
**Acute respiratory distress syndrome**												
No	36,175	34,943	1.00	< 0.001	2,086	32,388	1.00	< 0.001	6,103	30,072	1.00	< 0.001
Yes	5,364	435	2.30 (1.91–2.77)		2,244	2,888	12.22 (10.94–13.66)		2,483	2,881	4.51 (4.08–4.99)	
**Acute renal failure**												
No	36,796	34,848	1.00	< 0.001	2,972	32,119	1.00	< 0.001	6,428	30,368	1.00	< 0.001
Yes	4,239	218	4.59 (3.62–5.83)		904	3,169	2.72 (2.35–3.14)		2,146	2,093	2.92 (2.63–3.24)	
**Cardiovascular disease**												
No	37,830	46,801	1.00	< 0.001	3,208	32,278	1.00	0.005	5,742	32,088	1.00	< 0.001
Yes	27,659	10,064	1.53 (1.43–1.65)		2,570	23,547	1.16 (1.05–1.29)		9,234	18,425	1.32 (1.23–1.42)	
**Diabetes**												
No	50,393	51,815	1.00	< 0.001	4,301	43,131	1.00	0.055	10,053	40,340	1.00	< 0.001
Yes	15,096	5,050	1.38 (1.27–1.50)		1,477	12,694	1.12 (1.00–1.25)		4,923	10,173	1.23 (1.14–1.33)	
**Hypertension**												
No	50,186	50,433	1.00	< 0.001	4,827	42,477	1.00	0.005	10,212	39,974	1.00	0.017
Yes	15,303	6,432	1.40 (1.28–1.53)		951	13,348	0.81 (0.70–0.94)		4,764	10,539	0.90 (0.82–0.98)	
**Chronic lung disease**												
No	55,528	52,802	1.00	< 0.001	4,873	47,382	1.00	0.020	11,718	43,810	1.00	0.003
Yes	9,961	4,063	1.66 (1.52–1.82)		905	8,443	0.86 (0.75–0.98)		3,258	6,703	1.14 (1.04–1.24)	
**Chronic renal disease**												
No	63,236	55,809	1.00	< 0.001	5,612	53,879	1.00	0.022	14,038	49,198	1.00	< 0.001
Yes	2,253	1,056	1.38 (1.19–1.60)		166	1,946	0.75 (0.58–0.96)		938	1,315	1.47 (1.29–1.68)	
**Symptom onset to diagnosis**	89,631	107,713	0.99 (0.98–0.99)	< 0.001	7,705	75,913	1.00 (0.99–1.01)	0.471	19,194	70,437	0.95 (0.94–0.95)	< 0.001
**Healthcare worker**												
No	64,932	46,771	1.00	< 0.001	5,685	55,266	1.00	0.009	15,178	49,754	1.00	< 0.001
Yes	3,981	29,217	0.24 (0.22–0.26)		260	3,412	0.74 (0.60–0.93)		80	3,901	0.32 (0.23–0.47)	

CI: confidence interval; COVID-19: coronavirus disease; Hosp: hospitalised; ICU: admitted to intensive care unit; No ICU: not admitted to ICU; Non-hosp: Non-hospitalised; OR: odds ratio.

^a^ Only among hospitalised cases.

OR and 95% CI adjusted for sex; age group; Autonomous Region; presence of pneumonia and acute respiratory distress syndrome; diabetes; hypertension; cardiovascular, lung and renal diseases; days from symptom onset to diagnosis; healthcare worker; date of diagnosis and date of reporting.

Males had higher ORs of severe outcomes: hospitalisation (OR: 1.42; 95% CI: 1.34–1.50), ICU admission (OR: 1.36; 95% CI: 1.24–1.51) and death (OR: 1.33; 95% CI: 1.24–1.42) than females. Pneumonia was associated with a 27-fold higher odds of hospitalisation (OR: 26.63; 95% CI: 25.03–28.33). Patients who presented with cardiovascular disease were more likely to have a severe outcome. There was a strong association between ARDS and ICU admission (OR:  12.22; 95% CI: 10.94–13.66), while patients aged ≥ 80 years (OR: 0.12; 95% CI: 0.09–0.16) or with chronic renal disease (OR: 0.75; 95% CI: 0.58–0.96) had a lower odds of admission to ICU. Older age predicted mortality (p for trend < 0.001), with highest odds of death among patients ≥ 80 years (OR: 28.45; 95% CI: 19.85–40.78), compared with patients < 40 years. Looking at predisposing conditions, chronic kidney disease had the highest OR of death (OR: 1.47; 95% CI: 1.29–1.68).

Being a HCW was associated with a decreased risk of hospitalisation (OR: 0.24; 95%CI: 0.22–0.26) and death (OR: 0.32; 95% CI: 0.23–0.47). Each additional day from onset of symptoms to diagnosis was associated with a 1% and 5% reduction in the odds of hospitalisation and death, respectively.

Corresponding analyses by sex can be found in the supplementary material (Supplementary Tables S1, S2 and S3, respectively). Age, ARDS, cardiovascular disease or chronic lung disease increased the risk of hospitalisation more in males than in females (Supplementary Table S1). There were no differences in any risk factor for ICU admission and death when comparing males and females (Supplementary Table S3).

## Discussion

The first wave of the COVID-19 pandemic in Spain resulted in 218,652 cases reported to the RENAVE in a period of 9 weeks, from 31 January to 27 April. The national lockdown implemented on 15 March led to an inflection in the epidemic curve, as 6 days later the number of daily cases began to decrease. Although only 45% of the total reported COVID-19 cases were hospitalised during the study period, the healthcare system was overwhelmed and critical services were saturated, with 4.6% of cases admitted to ICUs and 11.9% dying.

From 31 January 2020, when the first imported COVID-19 case was detected in Spain, to 26 February, when the first case of local transmission was identified, only imported cases were notified. However, the retrospective identification of SARS-CoV-2 in swabs collected for other diagnoses or in hospitalised cases with pneumonia (unpublished data) confirmed that the epidemic had begun in Spain prior to this, though it had gone unnoticed. Moreover, the Spanish Influenza Sentinel Surveillance System [8] and the Sentinel Surveillance System in Catalonia [9] detected an unusual increase of weekly influenza-like illness cases at the beginning of March, suggesting that SARS-CoV-2 might have already been circulating in the population for several weeks. From the end of February, the number of cases increased exponentially, peaking on 20 March; this peak occurred 5 days after the implementation of the national lockdown, which is concordant with the reported median incubation time of COVID-19. During the following 6 weeks, there was a sustained decline in the number of COVID-19 cases, which evidenced the success of the public health interventions implemented to control the pandemic in Spain, similar to what has been reported previously in other countries [10].

The geographical distribution of COVID-19 varied greatly among the ARs. The cumulative incidence rate in the most affected region was 12 times higher than in the least affected AR. Similarly, the region of Lombardy in the north of Italy was more affected than the country’s central and southern areas [11]. These regional differences might be related to where the first outbreaks were declared [12], as some regions had to respond after the epidemic had already begun, while others had more time to prepare and take mitigation measures. Other factors—such as differences in mass social events, movements between regions before the lockdown [13], population densities and earlier silent transmission—might also have contributed to this heterogeneity.

The most frequently reported COVID-19 symptoms were fever, cough and shortness of breath, as shown in previous studies [14]. Gastrointestinal symptoms were also reported, more often in females than males, with 26.1% of patients experiencing diarrhoea and 8.4% vomiting. These proportions are higher than what was reported from China [15,16], but are similar to the results observed in a retrospective study from the US [17]. Sex differences for other mild symptoms—such as fatigue, anosmia, headache and sore throat—have been described as more frequent in females than in males [18]. Other symptoms, such as anosmia and ageusia, were not included in the notification form because their causal relationship with COVID-19 was unnoticed at the beginning of the pandemic and was highly debated until mid-April [19,20], probably because of their higher incidence in paucisymptomatic patients [21]. However, the Spanish sero-epidemiological study ENE-COVID identified anosmia as the most frequent COVID-19 symptom (40.2%) [22].

In Spain, the pandemic had a less severe impact on children and younger adults, with 62% of non-hospitalised patients < 60 years old. Furthermore, the number of severe and critical outcomes among these cases was low compared with older groups, which is consistent with data from Italy [23]. Similar to in other European countries, cases aged < 40 years represented a small proportion of COVID-19–related deaths in Spain [24,25], with only five deaths in children aged < 15 years in our cohort, four of whom had underlying conditions.

The bimodal distribution of non-hospitalised cases indicates that many elderly cases were not admitted to hospitals. Moreover, patients aged ≥ 80 years had the lowest probability of ICU admission. This is probably because of the high pressure on the healthcare system, which may have precluded the hospitalisation of critically ill elderly people or those with particular clinical presentations in some ARs. Also, ICU beds were probably prioritised for younger age groups because of the invasive nature of the treatments in the ICU and the poor prognosis for older patients.

Elderly cases suffered the greatest mortality burden. Age was the most important risk factor for death and patients ≥ 80 years old were 28 times more likely to die than those < 40 years old. Nursing homes were severely hit by the pandemic in Spain [26-28], as seen in other countries [29-31], which highlights the need to prioritise implementing a strategy for the surveillance and prevention of COVID-19 in long-term care facilities in European countries [30] in order to gain control of the pandemic. The overall case fatality rate was 11.9%, lower than in Italy (14%) [11], but far higher than in China (3.8%) [32] or Germany (4.7%) [33]. These differences might be partially explained by different testing and surveillance strategies, which were initially focused on severe cases in Spain, as well as by the different age distribution of the Spanish population; the median age of cases in Spain was 61 years old, which was similar to in Italy [11] but higher than in China (47 years old) [16].

In this study, we found sex differences in the number of COVID-19 cases and disease severity. Overall, there were more female cases, which could be attributed to the higher proportion of female HCWs, but also to the higher proportion of females aged ≥ 80 years in the overall population [7]. On the other hand, males had a higher risk of severe outcomes (hospitalisation, ICU admission and death), similar to that observed in other countries [34-36]. However, there are few studies on risk factors for severity disaggregated by sex [37]. In our study, mortality was higher in males, but we could not find any factor that modified the risk of death differently in males than in females. As Falahi suggests [38], females could be less susceptible to complications related to viral infections based on a different innate immunity, steroid hormones or factors related to sex chromosomes. Also, a higher expression of the angiotensin converting enzyme (ACE) 2 as a viral receptor in men than in women, makes men more susceptible to SARS-CoV-2 infection and to more severe clinical outcomes than women [39,40].

Some authors have postulated an association between ACE inhibitors or angiotensin receptor blockers (ARBs) and the increased severity of the disease as an explanation for the association with hypertension and poorer prognosis [41,42]. In this analysis, the prevalence of hypertension was two-fold greater among hospitalised cases than non-hospitalised cases (23.4% and 11.3%, respectively), but it was not found as a risk factor for ICU admission or death.

Patients with pneumonia had a higher odds of hospitalisation, while the strongest predictor for admission to ICU was the development of ARDS, similar to what was found in a study in Vitoria [43]. Underlying diseases were common among cases, especially among males; the vast majority of those who died had at least one. Some of these comorbidities were found to be associated with disease severity. As in previous studies, diabetes was associated with an increased risk of hospitalisation and death [44]. Chronic renal disease was not associated with ICU admission, though it was the comorbidity with the highest risk of death and its association with mortality has been highlighted elsewhere [45]. Cardiovascular disease was a consistent risk factor across all severe outcomes [46], even though it was not the strongest one.

Remarkably, 22.9% of the cases were HCWs, a fraction substantially higher than that reported in China (3.8%) [44] and Italy (12.2%) [11], and probably one of the highest in Europe. The high proportion of HCW cases may reflect the testing strategy among essential workers, although the lack of personal protective equipment, especially during the first weeks of the COVID-19 epidemic, may have contributed to such a high rate of infection [48]. Despite this, HCWs were less likely to be hospitalised and die compared with other COVID-19 cases (0.6% vs 12%). Further studies are needed to compare the severity of COVID-19 in HCWs compared with the general population.

This analysis may be subject to some limitations. During the COVID-19 epidemic peak in Spain, response capacities were strained and protocols had to be adapted to prioritise testing for severe cases and essential workers. Consequently, most of the mild and asymptomatic cases were neither confirmed nor notified to health authorities. Only confirmed cases were used in this analysis, resulting in an underestimation of COVID-19 cases in the population. When the testing capacity increased, a proportion of them were notified retrospectively, with a significant lag in reporting since their onset of symptoms. The overload of notifications received by local public health services during the most intense weeks of the COVID-19 epidemic also led to incomplete or minimised data collection of some variables, especially those on underlying health conditions, which may have under/over-estimated some associations. In addition, some variables were not clearly defined, such as HCW, which included a heterogeneous group of professionals because of different regions’ interpretations.

Despite these challenges, the regional public health services put immense effort into reporting the best possible case-based information. The exhaustive data collected, owing to their collaboration, resulted in one of the largest COVID-19 datasets in Europe and allowed for an extensive analysis of the COVID-19 cases in Spain. Using the wide spectrum of variables and the precise information of this dataset, we were able to characterise COVID-19 cases, describe patterns and identify risk factors for disease severity in this population. This work represents one of the few analyses that examined the characteristics of COVID-19 in males and females separately. The long period selected for this analysis provided a full picture of the course of the first wave of the COVID-19 pandemic in Spain, starting before the implementation of a national lockdown and ending at the start of the first de-escalation phase, allowing for a broader understanding of the impact of key public health control measures and events.

In most European countries, transmission of SARS-CoV-2 was reduced after the implementation of multiple response measures, including stay-at-home recommendations for high-risk groups or the general population. There was substantial heterogeneity in the public health measures implemented across countries [10]. In Spain, although several initial social distancing measures—as well as school closures—were implemented during the first weeks of March, the national lockdown was the most important measure that mitigated the COVID-19 epidemic, as 5 days after the lockdown the number of COVID-19 cases sharply decreased. This observation might help to guide decisions on the most suitable public health measures that could be adopted in future waves of the COVID-19 pandemic in Spain.

### Conclusions

Spain experienced one of the worst first waves of the COVID-19 pandemic in Europe and the world, lasting for a period of 2 months from the confirmation of local transmission. The national lockdown managed to stop the COVID-19 epidemic curve from rising and to mitigate SARS-CoV-2 transmission. This descriptive and analytical report of the first 218,652 COVID-19 cases in Spain highlights the pandemic’s impact on the elderly population and the high rates of infection in HCWs, while also outlining independent risk factors for disease severity and mortality. Continued and standardised surveillance of COVID-19 is crucial in order to provide the best possible information to guide local, state and international authorities and to move forward towards control of the pandemic and prevention of future outbreaks.

## Members of the Working group for the surveillance and control of COVID-19 in Spain

Lidia Redondo-Bravo, María José Sierra Moros (Coordination Centre for Health Alerts and Emergencies, Spanish Ministry of Health, Madrid);

Elena Vanessa Martínez Sánchez (Coordination Centre for Health Alerts and Emergencies, Spanish Ministry of Health, Madrid and CIBER Epidemiologia y Salud Pública (CIBERESP));

Nicola Lorusso, Alberto Carmona Ubago, Virtudes Gallardo García (Dirección General de Salud Pública y Ordenación Farmacéutica, Sevilla, Andalucía);

Pilar Sánchez Villanueva (Sección Vigilancia Teruel, Aragón);

Adela Puy Azón (Sección Vigilancia Huesca, Aragón);

Joaquín Guimbao Bescós (Sección Vigilancia Zaragoza, Aragón);

An LD Boone, Ana Fernández Ibáñez, Blanca Álvarez Fernández (Servicio de Vigilancia Epidemiológica, Dirección General de Salud Pública, Asturias);

Antonio Nicolau Riutort, Magdalena Salom Castell, Jaume Giménez Duran (Direcció General Salut Pública i Participació, Illes Balears);

Domingo Núñez Gallo, Magdalena Lucia Rojo Moreno (Dirección General de Salud Pública, Islas Canarias);

Aniceto Blasco de la Fuente, Luis Javier Viloria Raymundo (Dirección General Salud Pública, Cantabria);

Marcos Hernández Pereña (Unidad Docente Medicina Preventiva y Salud Pública, Cantabria);

Matilde Chico Mena (Sección Epidemiología Ciudad Real, Castilla-La Mancha);

Sonia Humanes Aparicio (Servicio Epidemiología, Castilla-La Mancha);

Soledad Illescas Fernández-Bermejo (Servicio de Microbiología, Hospital General Universitario Ciudad Real, Castilla-La Mancha);

Socorro Fernández Arribas, Mª del Henar Marcos Rodríguez (Servicio de Epidemiología, Dirección General de Salud Pública, Castilla y León);

Isabel Martínez-Pino (Servicio de Epidemiología, Dirección General de Salud Pública, Castilla y León and CIBER Epidemiologia y Salud Pública (CIBERESP));

Mireia Jané, Ana Martínez, Pilar Ciruela (Subdirección General de Vigilancia y Respuesta a Emergencias de Salud Pública, Agencia de Salud Pública, Catalunya and CIBER Epidemiologia y Salud Pública (CIBERESP));

Katja Villatoro Bongiorno, Aina March Yagüe, Jordi Pérez Panadés (Servicio de Vigilancia y Control Epidemiológico, Dirección General de Salud Pública y Adicciones, Comunidad Valenciana);

María del Mar López-Tercero Torvisco, Cecilia Gordillo Romero, Beatriz Caleya Olivas (Dirección General de Salud Pública, Extremadura);

Sara De Miguel García, Esther Córdoba Deorador, Elisa Gil Montalbán (Subdirección General de Epidemiología, Dirección General de Salud Pública, Comunidad de Madrid);

Manuel del Valle Arrojo (Epidemiología A Coruña, Galicia);

Luisa Abraira García (Epidemiología Lugo, Galicia);

Antonio Boullosa Cortés (Epidemiología Pontevedra, Galicia);

Ana García-Fulgueiras, Mª Dolores Chirlaque, Mª Isabel Barranco (Servicio de Epidemiología, Dirección General de Salud Pública y Adicciones, IMIB-Arrixaca, Murcia and CIBER Epidemiologia y Salud Pública (CIBERESP));

Itziar Casado, Jesús Castilla, Manuel García-Cenoz (Instituto de Salud Pública de Navarra - IdiSNA, Pamplona and CIBER Epidemiologia y Salud Pública (CIBERESP));

Fernando Gonzalez Carril, Amaia Soraluce Olañeta, Mª Jesús Lázaro-Carrasco de la Fuente (Servicio de Vigilancia y Vacunas, Dirección de Salud Pública y Adicciones, País Vasco);

Eva Martínez Ochoa, Ana Carmen Ibáñez Pérez, Ángela Blanco Martínez (Servicio de Epidemiología y Prevención Sanitaria, Dirección General de Salud Pública, Consumo y Cuidados, La Rioja);

Ana Isabel Rivas Pérez, Violeta Ramos Marín, Margarita Medina Vinuesa (Servicio de Epidemiología, Consejería de Sanidad, Consumo y Gobernación, Ceuta);

Daniel Castrillejo Pérez, Atanasio Alfonso Gómez Anés (Servicio de Epidemiología, Dirección General de Salud Pública y Consumo, Melilla);

Francisco Pozo, Inmaculada Casas (National Centre of Microbiology, CIBER Epidemiologia y Salud Pública (CIBERESP), Carlos III Health Institute, Majadahonda);

Belén Peñalver-Argüeso (Unidad Docente Medicina Preventiva y Salud Pública, Escuela Nacional de Sanidad, Carlos III Health Institute, Madrid);

Despina Pampaka (European Programme for Intervention Epidemiology Training (EPIET), European Centre for Disease Prevention and Control (ECDC), Stockholm, Sweden and National Centre of Epidemiology, Carlos III Health Institute, Madrid) and

Jesús A. Oliva Domínguez, María Sastre García, Amparo Larrauri (National Centre of Epidemiology and CIBER Epidemiologia y Salud Pública (CIBERESP), Carlos III Health Institute, Madrid).

## Supplementary Data

565000 Supplementary materials
