# Supplementary material for: The first wave of the COVID-19 pandemic in Spain: characterisation of cases and risk factors for severe outcomes, as at 27 April 2020
Source: Euro Surveill. 2020 Dec 17;25(50):2001431. doi: 10.2807/1560-7917.ES.2020.25.50.2001431 (PMC7812423; doi:10.2807/1560-7917.ES.2020.25.50.2001431)
Supplement: Supplementary materials [file 20-01431_LARRAURI_supplement.pdf]

"This supplementary material is hosted by *Eurosurveillance* as supporting information alongside the article “The COVID-19 epidemic in Spain: Characterisation of cases and risk factors for severe outcomes”, on behalf of the authors, who remain responsible for the accuracy and appropriateness of the content. The same standards for ethics, copyright, attributions and permissions as for the article apply. Supplements are not edited by *Eurosurveillance* and the journal is not responsible for the maintenance of any links or email addresses provided therein."

Tables

Table S1. Analysis of factors associated with hospitalisation, by sex.

	Females				Males				
	Hosp.	Non-hosp.	OR (95%CI)	*p-val*	Hosp.	Non-hosp.	OR (95%CI)	*p-val*	*p-int*
Age group (years)									
<40	2955	17178	1.00		2983	8622	1.00		
40-59	9450	26926	1.30 (1.15-1.46)	<0.001	13382	14118	1.69 (1.48-1.93)	<0.001	
60-69	6743	6842	2.32 (2.02-2.68)	<0.001	10104	5083	3.42 (2.93-3.99)	<0.001	
70-79	8218	4286	4.01 (3.41-4.70)	<0.001	11899	3721	5.33 (4.49-6.32)	<0.001	
≥80	12458	15340	3.01 (2.59-3.50)	<0.001	11431	5575	4.34 (3.65-5.16)	<0.001	<0.001
p-trend				<0.001				<0.001	
Pneumonia									
No	5636	25318	1.00		5376	12240	1.00		
Yes	24001	3256	27.30 (25.10-29.68)	<0.001	32430	2732	26.17 (23.85-28.71)	<0.001	0.079
Acute respiratory distress syndrome									
No	16517	23172	1.00		19658	11771	1.00		
Yes	1928	269	1.69 (1.32-2.15)	<0.001	3436	165	3.40 (2.53-4.56)	<0.001	<0.001
Acute renal failure									
No	16752	23125	1.00		20044	11722	1.00		
Yes	1556	121	4.68 (3.38-6.49)	<0.001	2683	97	4.46 (3.14-6.34)	<0.001	0.792
Cardiovascular disease									
No	17459	31439	1.00		20370	15361	1.00		
Yes	11354	6115	1.49 (1.36-1.65)	<0.001	16305	3949	1.57 (1.42-1.75)	<0.001	0.001
Diabetes									
No	22760	34546	1.00		27632	17268	1.00		
Yes	6053	3008	1.38 (1.23-1.54)	<0.001	9043	2042	1.38 (1.22-1.56)	<0.001	0.151
Hypertension									

No	21582	33376	1.00		28603	17056	1.00		
Yes	7231	4178	1.46 (1.29-1.64)	<0.001	8072	2254	1.34 (1.17-1.54)	<0.001	0.969
Chronic lung disease									
No	25317	35143	1.00		30210	17658	1.00		
Yes	3496	2411	1.49 (1.31-1.70)	<0.001	6465	1652	1.81 (1.59-2.07)	<0.001	0.002
Chronic renal disease									
No	27846	36859	1.00		35389	18949	1.00		
Yes	967	695	1.29 (1.06-1.57)	0.012	1286	361	1.51 (1.20-1.90)	<0.001	0.031
Symptom onset to diagnosis	39827	70579	0.99 (0.98-0.99)	<0.001	49803	37133	0.98 (0.98-0.99)	<0.001	0.306
Health care worker									
No	28009	27983	1.00		36922	18787	1.00		
Yes	2632	22693	0.24 (0.21-0.26)	<0.001	1349	6524	0.23 (0.20-0.26)	<0.001	0.103

OR and 95%CI adjusted for sex, age group, autonomous region, presence of pneumonia and acute respiratory distress syndrome, diabetes, hypertension, cardiovascular, lung and renal diseases, days between symptom onset until diagnosis and health care work. *Only among hospitalised cases.

Table S2. Analysis of factors associated with admission to ICU, by sex.

	Females				Males				
	ICU	Non-ICU	OR (95%CI)	*p-val*	ICU	Non-ICU	OR (95%CI)	*p-val*	*p-int*
Age group (years)									
<40	145	2618	1.00		246	2566	1.00		
40-59	647	8151	1.29 (0.89-1.85)	0.175	1611	10897	1.34 (1.02-1.75)	0.037	
60-69	720	5543	1.77 (1.23-2.57)	0.002	1776	7690	1.81 (1.37-2.39)	<0.001	
70-79	688	7013	1.41 (0.96-2.05)	0.077	1509	9702	1.17 (0.88-1.56)	0.280	
≥80	167	11258	0.13 (0.08-0.21)	<0.001	196	10471	0.10 (0.07-0.15)	<0.001	0.153
p-trend				<0.001				<0.001	
Pneumonia									
No	210	4908	1.00		345	4561	1.00		
Yes	1735	20655	1.33 (1.06-1.68)	0.013	4025	26484	1.61 (1.34-1.93)	<0.001	0.349
Acute respiratory distress syndrome									
No	690	15043	1.00		1396	17345	1.00		
Yes	638	1178	14.40 (11.84-17.52)	<0.001	1606	1710	11.43 (9.98-13.08)	<0.001	0.060
Acute renal failure									

No	988	14967	1.00		1984	17152	1.00		
Yes	204	1280	1.93 (1.47-2.55)	<0.001	700	1889	3.11 (2.62-3.70)	<0.001	0.126
Cardiovascular disease									
No	1034	15312	1.00		2174	16965	1.00		
Yes	703	9982	1.23 (1.02-1.47)	0.029	1867	13565	1.13 (0.99-1.28)	0.066	0.248
Diabetes									
No	1346	20028	1.00		2955	23102	1.00		
Yes	391	5266	1.07 (0.87-1.31)	0.542	1086	7428	1.15 (1.01-1.33)	0.042	0.993
Hypertension									
No	1447	18836	1.00		3380	23640	1.00		
Yes	290	6458	0.81 (0.62-1.04)	0.094	661	6890	0.81 (0.68-0.97)	0.022	0.313
Chronic lung disease									
No	1488	22277	1.00		3385	25104	1.00		
Yes	249	3017	0.95 (0.75-1.21)	0.680	656	5426	0.82 (0.70-0.96)	0.016	0.127
Chronic renal disease									
No	1679	24451	1.00		3933	29427	1.00		
Yes	58	843	0.97 (0.64-1.48)	0.895	108	1103	0.65 (0.47-0.88)	0.006	0.051
Symptom onset to diagnosis	2367	34585	1.00 (0.98-1.01)	0.852	5338	41327	1.00 (0.98-1.01)	0.432	0.179
Health care worker									
No	1697	24518	1.00		3988	30747	1.00		
Yes	108	2317	0.71 (0.51-0.99)	0.045	152	1095	0.82 (0.60-1.11)	0.190	0.196

OR and 95%CI adjusted for sex, age group, autonomous region, presence of pneumonia and acute respiratory distress syndrome, diabetes, hypertension, cardiovascular, lung and renal diseases, days between symptom onset until diagnosis and health care work. *Only among hospitalised cases.

Table S3. Analysis of factors associated with death, by sex.

	**Females**				**Males**				
	Dead	Alive	OR (95%CI)	*p-val*	Dead	Alive	OR (95%CI)	*p-val*	*p-int*
Age group (years)									
<40	43	2912	1.00		61	2922	1.00		
40-59	318	9132	1.99 (1.10-3.58)	0.023	703	12679	1.93 (1.20-3.10)	0.007	
60-69	606	6137	4.04 (2.27-7.20)	<0.001	1496	8608	5.36 (3.36-8.55)	<0.001	
70-79	1628	6590	8.16 (4.62-14.41)	<0.001	3498	8401	11.26 (7.08-17.92)	<0.001	
≥80	5071	7387	23.91	<0.001	5770	5661	31.46	<0.001	0.271

			(13.57-42.13)				(19.75-50.12)		
p-trend				<0.001				<0.001	
Pneumonia									
No	966	4670	1.00		1062	4314	1.00		
Yes	4931	19070	1.23 (1.08-1.40)	0.002	7883	24547	1.25 (1.11-1.41)	<0.001	0.678
Acute respiratory distress syndrome									
No	2546	13971	1.00		3557	16101	1.00		
Yes	884	1044	4.95 (4.20-5.84)	<0.001	1599	1837	4.25 (3.74-4.83)	<0.001	0.538
Acute renal failure									
No	2692	14060	1.00		3736	16308	1.00		
Yes	757	799	2.62 (2.22-3.10)	<0.001	1389	1294	3.15 (2.76-3.59)	<0.001	0.105
Cardiovascular disease									
No	2418	15041	1.00		3324	17046	1.00		
Yes	3487	7867	1.37 (1.22-1.53)	<0.001	5747	10558	1.28 (1.16-1.41)	<0.001	0.814
Diabetes									
No	4063	18697	1.00		5990	21642	1.00		
Yes	1842	4211	1.24 (1.10-1.39)	<0.001	3081	5962	1.23 (1.11-1.35)	<0.001	0.966
Hypertension									
No	3738	17844	1.00		6474	22129	1.00		
Yes	2167	5064	0.89 (0.77-1.02)	0.094	2597	5475	0.91 (0.81-1.03)	0.128	0.964
Chronic lung disease									
No	4960	20357	1.00		6758	23452	1.00		
Yes	945	2551	1.13 (0.97-1.30)	0.118	2313	4152	1.14 (1.03-1.27)	0.013	0.814
Chronic renal disease									
No	5523	22323	1.00		8515	26874	1.00		
Yes	382	585	1.43 (1.17-1.74)	<0.001	556	730	1.51 (1.27-1.80)	<0.001	0.830
Symptom onset to diagnosis	7666	32161	0.94 (0.93-0.95)	<0.001	11528	38275	0.95 (0.94-0.96)	<0.001	0.514
Health care worker									
No	5980	22029	1.00		9198	27724	1.00		
Yes	27	2605	0.21 (0.11-0.39)	<0.001	53	1296	0.42 (0.27-0.66)	<0.001	0.106

OR and 95%CI adjusted for sex, age group, autonomous region, presence of pneumonia and acute respiratory distress syndrome, diabetes, hypertension, cardiovascular, lung and renal diseases, days between symptom onset until diagnosis and health care work. *Only among hospitalised cases.

Figures

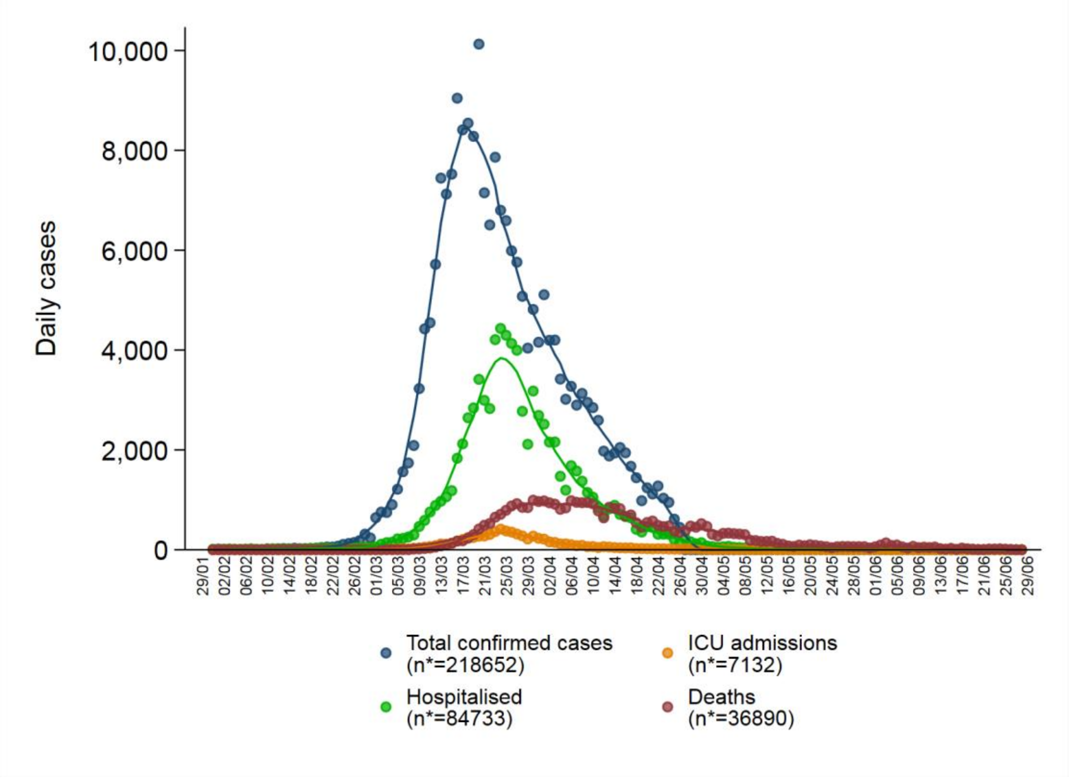

Figure S1. Epidemic curve of COVID-19 confirmed cases by severity group. Spain, January-April 2020. RENAVE. Spain, January-April 2020.

COVID-19 confirmed cases by date of onset of symptoms or date of diagnosis minus 6 days; hospitalised cases by hospitalisation date; ICU admitted cases by admission date; deaths by date of death.

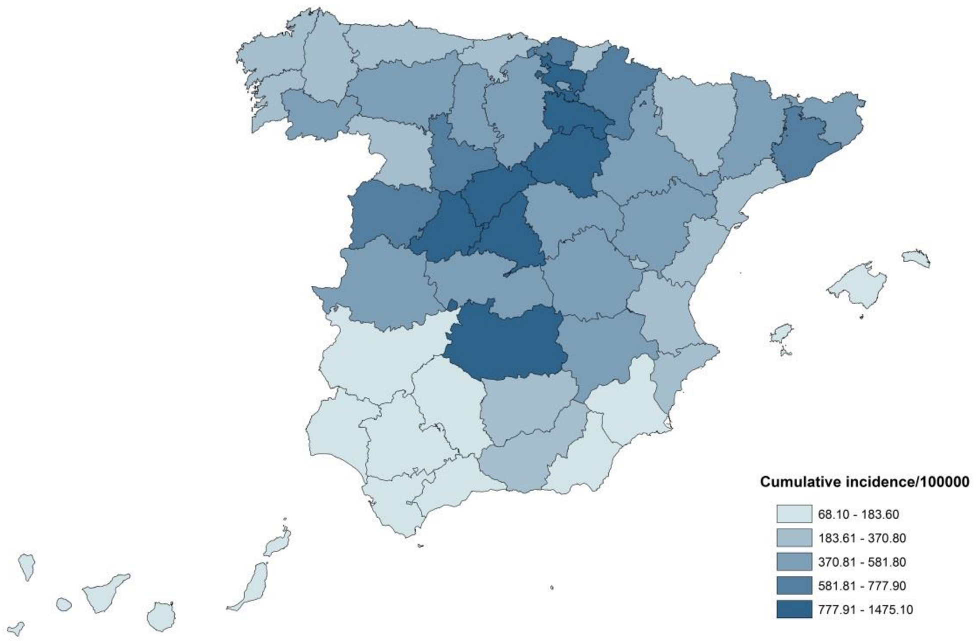

Figure S2. Age-adjusted cumulative incidence (per 100 000 inhabitants) of COVID-19 cases, per province. RENAVE. Spain, January-April 2020
